# A New Nanocomposite Packaging Based on LASiS-Generated AgNPs for the Preservation of Apple Juice

**DOI:** 10.3390/antibiotics10070760

**Published:** 2021-06-22

**Authors:** Maria Chiara Sportelli, Antonio Ancona, Annalisa Volpe, Caterina Gaudiuso, Valentina Lavicita, Valerio Miceli, Amalia Conte, Matteo Alessandro Del Nobile, Nicola Cioffi

**Affiliations:** 1 Chemistry Department, University of Bari, Via Orabona 4, 70126 Bari, Italy; maria.sportelli@uniba.it; 2 Physics Department, Institute of Photonics and Nanotechnology—National Research Council (IFN-CNR), University of Bari, Via Amendola 173, 70126 Bari, Italy; antonio.ancona@uniba.it (A.A.); annalisa.volpe@uniba.it (A.V.); caterina.gaudiuso@uniba.it (C.G.); 3 Physics Department, University of Bari, Via Orabona 4, 70126 Bari, Italy; 4 Department of Agricultural Sciences, Food and Environment, University of Foggia, Via Napoli 25, 71122 Foggia, Italy; valentina.lacivita@unifg.it (V.L.); amalia.conte@unifg.it (A.C.); matteo.delnobile@unifg.it (M.A.D.N.); 5 Ente per le Nuove Tecnologie, l’Energia e l’Ambiente (ENEA) Research Center, BIOAG Division-ss Appia km 700, 72100 Brindisi, Italy; valerio.miceli@enea.it

**Keywords:** nanoantimicrobials, silver nanoparticles, laser ablation synthesis in solution, poly(-3-hydroxybutyrate-co-3-hydroxyvalerate), sustainable active packaging

## Abstract

Designing bioactive materials, with controlled metal ion release, exerting a significant biological action and associated to low toxicity for humans, is nowadays one of the most important challenges for our community. The most looked-for nanoantimicrobials are capable of releasing metal species with defined kinetic profiles, either by slowing down or inhibiting bacterial growth and pathogenic microorganism diffusion. In this study, laser ablation synthesis in solution (LASiS) has been used to produce bioactive Ag-based nanocolloids, in isopropyl alcohol, which can be used as water-insoluble nano-reservoirs in composite materials like poly(3-hydroxybutyrate-co-3-hydroxyvalerate). Infrared spectroscopy was used to evaluate the chemical state of pristine polymer and final composite material, thus providing useful information about synthesis processes, as well as storage and processing conditions. Transmission electron microscopy was exploited to study the morphology of nano-colloids, along with UV-Vis for bulk chemical characterization, highlighting the presence of spheroidal particles with average diameter around 12 nm. Electro-thermal atomic absorption spectroscopy was used to investigate metal ion release from Ag-modified products, showing a maximum release around 60 ppb, which ensures an efficient antimicrobial activity, being much lower than what recommended by health institutions. Analytical spectroscopy results were matched with bioactivity tests carried out on target microorganisms of food spoilage.

## 1. Introduction

Due to the overwhelming presence of plastics in the environment, the use of biodegradable polymers is preferred in respect to petroleum-based plastics which are, although recyclable, highly polluting and difficult to waste [1]. In this panorama, the research is focused towards the use of low-cost, ecofriendly materials which are able to still ensure rheological and mechanical properties compatible with those of common plastics [2]. Food industry is moving fast in this direction; many biodegradable materials are used as food packaging nowadays: cellulose- and hemp-based materials, polylactic acid (PLA), etc. [3]. Among biopolymers, polyhydroxyalkanoates (PHAs) are a group of polyesters synthesized by more than 300 species of Gram-positive and Gram-negative bacteria. Thanks to their thermoplastic properties similar to those of common polyolefins, PHAs are good candidates to substitute petroleum plastics [4]. Food technology research has posed great interest in the use of a specific PHA: poly(3-hydroxybutyrate-co-3-hydroxyvalerate) (PHBV); in fact, it possesses high biocompatibility with slow hydrolytic degradation, and this makes PHBV biopolymer a perfect candidate for food packaging and biomedical applications [5]. The first PHA-based product approved by the Food and Drug Administration (FDA) for real-life application is “TephaFLEX”, an absorbable suture prepared from PHBV [6]. In 2007, the FDA had authorized its marketing in the USA [7], paving the way for the application of PHAs (and PHBV, specifically) in many different fields.

Many green processes have been developed in the last few years to produce PHBV (and other PHAs) with a low environmental impact, through fermentation of by-products and wastes from food industries. These processes do not need neither the use of pure bacterial cultures, nor a sterile environment, thus making them extremely appealing from an industrial and large-scale point of view. As an example, common dairy by-products like ricotta cheese, exhausted whey (RCEW) and buttermilk were recently used to produce PHBV by fermentation from halophile microorganisms [8].

The incorporation of nanoparticles into polymers is a promising way to confer them antimicrobial properties and/or to improve their barrier and mechanical characteristics [9,10]. Many different methods have been proposed in the last years for the preparation of polymeric composite materials, like tri-axial [11] and side-by side electrospinning [12], spraying [13], and solution casting [14]. Up to the last decade, food packaging was considered as a passive barrier to postpone the effect of degradation over the packaged product; however, nowadays propensities in chemistry and material science include the development of packaging materials, which can actively interact with both the environment and food, even playing an active role in its conservation [9,15]. Various hybrid materials were found effective against spoilage microorganisms, depending on the hydrophilic nature of the polymeric matrix and on boundary conditions, such as pH and ionic strength of the release solution [12]. However, to the best of our knowledge, no biopolymers based on PHAs have been involved in the development of active nanocomposite systems. 

Laser ablation synthesis in solution (LASiS) is a novel nanoparticle production route, which offers several benefits. First, it is green and straightforward, and it is based on the controlled disruption of a proper target by a high-energy laser beam, while it is immersed in solution. LASiS does not require neither reducing agents nor precursors. Moreover, capping agents are not mandatorily required, since the solvent itself can offer a modest stabilizing effect on as-prepared nanophases [16]. Several experimental parameters, including laser operating conditions, can be used to tune the features of the produced nanomaterial. Finally, the method has been proven to be suitable for the large-scale production of nanomaterials [17].

In this paper, we propose a biodegradable nanocomposite for active food packaging applications, based on silver nanoparticles (AgNPs) combined with PHBV. We chose a laser ablation synthesis in solution (LASiS) for the production of AgNPs; this method allows producing metal NPs in the absence of toxic reducing agents and free of stabilizing agents, thus making them suitable for food packaging and biomedical applications [15,18]. 2-propanol (IPA) was used as ablation medium, because it provides very stable colloids [16] and proved to be not affecting PHBV physicochemical properties. Both AgNPs and the corresponding AgNPs-PHBV composites were here characterized by means of different analytical techniques. Kinetics of antimicrobial ionic release were also studied in physiological environment by means of electro-thermal atomic absorption spectroscopy (ETAAS). AgNPs-PHBV composites were also tested on a food-simulating system.

## 2. Results and Discussion

### 2.1. LASiS of AgNPs and Preparation of AgNPs-PHBV Composites

AgNPs were obtained by ns-pulsed laser ablation in pure IPA, following the experimental procedure already reported in [15]. The laser ablation yield was about 0.11 ± 0.05 mg/mL.

**Figure 1:**
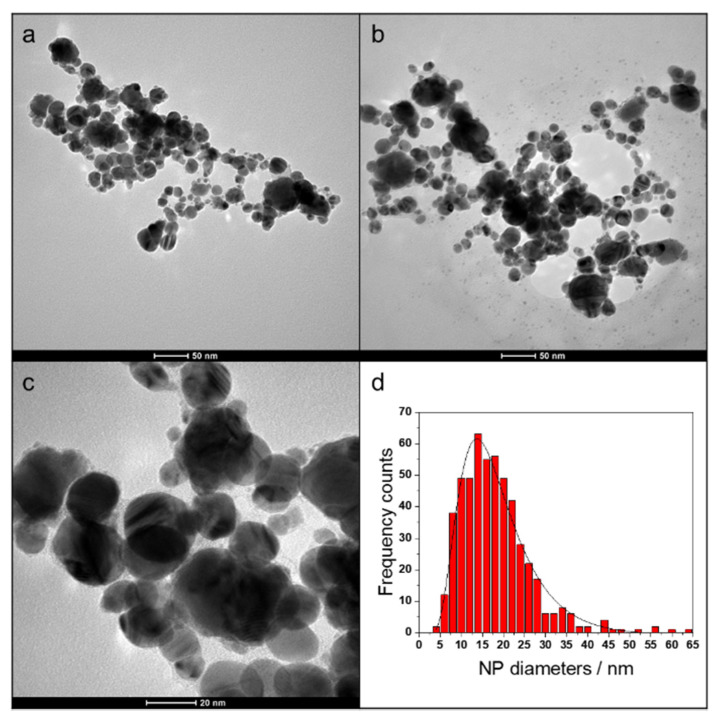
AgNPs prepared by LASiS (**a**–**c**) and their size distribution histogram (**d**).

AgNPs showed a spherical morphology (Figure 1), and a mono-modal size distribution with an average size diameter of 12 ± 6 nm (Figure 1c). NPs were surrounded by a thin organic shell, ascribable to the low-contrast halo around dark particles. As already reported in the literature, IPA goes through oxidizing and radicalization processes when exposed to high pressures and temperatures during LASiS synthesis, forming branched ketones and aldehydes, which are adsorbed on the NP surface [16]. High-magnification micrographs (Figure 1c) underscored the presence of interference fringes, related to NP crystallinity.

UV-Vis spectrum of a fresh colloid (Figure S1) reported, as expected, a surface plasmon resonance band centered at 404 ± 1 nm.

AgNPs-PHBV composite was prepared by simple drop-casting of the colloidal solution on PHBV sheets. Modified PHBV substrates were characterized spectroscopically, in order to understand whether the modification with AgNPs altered the polymer surface chemistry.

**Figure 2:**
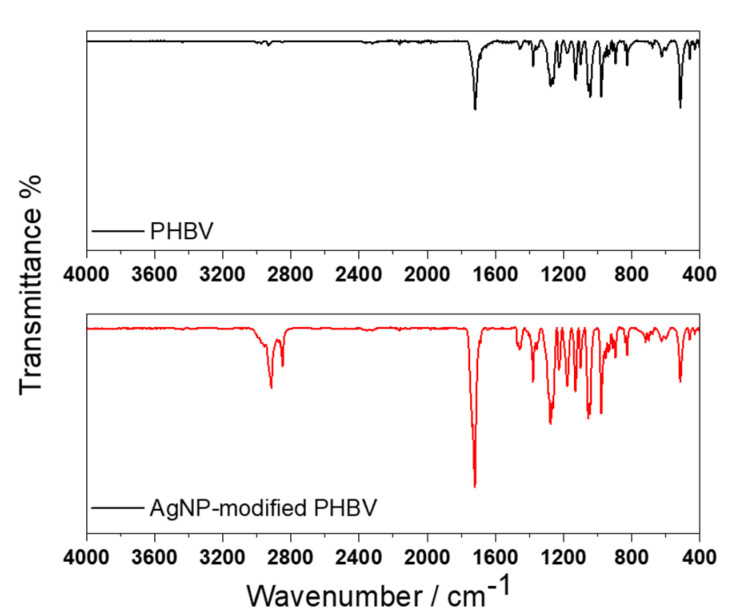
ATR-IR spectra of pristine (black line) and AgNP-modified (red line) PHBV samples.

Both a pristine and Ag-modified PHBV sheets were analyzed by attenuated total reflectance infrared (ATR-IR) spectroscopy. The two obtained spectra are reported in Figure 2. It is evident, comparing the two IR profiles, that the main absorption bands are in common between the two samples. The attribution of these bands is shown in Table 1.

The higher band intensity highlighted for the peak was at about 1718 cm^−1^, relevant to the carbonyl functionality, for the Ag-modified sample, is ascribable to the presence of the organic layer stabilizing NPs. The functional groups identified on the sample are in agreement with what was reported in the literature [19].

### 2.2. Kinetics of Silver Ions Release

**Figure 3:**
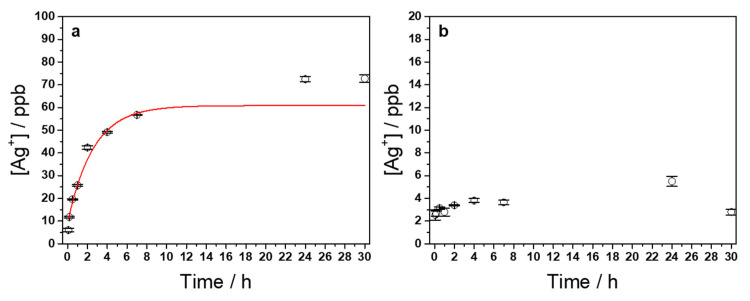
Silver ions release from AgNPs-PHBV composite film in PBS contact solution (**a**); R^2^ was equal to 0.9887. Corresponding control experiment on bare PHBV (**b**).

The investigation of the metal releasing properties was carried out evaluating the silver release kinetic curves from AgNPs-PHBV nanocomposites into phosphate-buffered saline solution (PBS) (Figure 3). The chosen timespan for this experiment was 30 h. A pristine PHBV sheet was used as control. For the Ag-modified sample (Figure 3a), the release kinetics followed an expected pseudo-first order model [20], as per Equation (1):

(1) $$y=y_{0}+a\left(1-e^{-bx}\right)$$

The plateau value of silver ions concentration was reached in about 3 h, and resulted equal to 61 ± 3 ppb. Kinetic constant (i.e., *b*) was calculated to be 2.8 ± 0.6 h^−1^. Silver ion concentration at time t = 0 (i.e., *y_0_*) was 7 ± 2 ppb. The presence of immediately soluble silver species (almost negligible), which are released in PBS as soon as the samples go in contact with the aqueous solution, are probably ascribable to a surface partial oxidation of AgNPs. This phenomenon was already reported for similar composite materials [15,18]. The highest released silver ion concentration, although active against food spoilage (vide infra), was much lower than the maximum one reported to be human-safe by the WHO (7 ppm) [21,22] and European authorities. ESFA (European Food Safety Authority) provided upper limits of Ag migration from packaging, in 2011. Recommendations are not to exceed 0.05 mg/L in water and 0.05 mg/kg in food [23]. A negligible release, not following any specific kinetic, and below 6 ppb, was found for the control sample (Figure 3b). Traces of metals on blank samples could be ascribed to metal plates used in thermoforming processes. No entire NP release was registered from the Ag-modified PHBV: ETAAS contact solution was imaged by TEM after 30 h of soaking. These micrographs only showed the presence of some salts and other impurities (Figure S3); no structures similar to LASiS AgNPs were visible.

### 2.3. Antimicrobial Effects of AgNPs-PHBV

**Figure 4:**
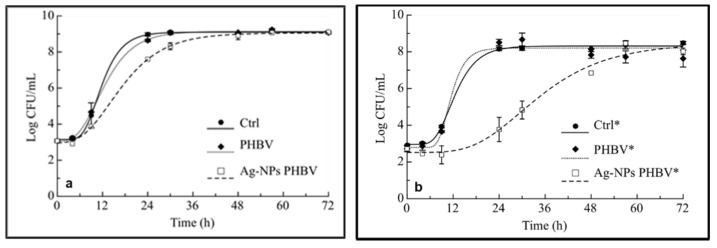
Evolution of *Pseudomonas* mix (*P. fluorescens* and *P. putida*) plotted during time. (**a**) Ctrl: bare inoculated sample; PHBV: inoculated sample with PHBV (4 cm^2^); AgNPs-PHBV: inoculated sample with AgNPs-PHBV (4 cm^2^). (**b**) Ctrl*: bare inoculated sample; PHBV*: inoculated sample with PHB (8 cm^2^); AgNPs-PHBV*: inoculated sample with AgNPs-PHBV (8 cm^2^).

The antimicrobial efficacy of AgNPs-PHBV composites was tested in a food simulating system (clear apple juice) inoculated with a mix of *Pseudomonas*, which are considered foodborne bacteria [24,25]. Results recorded during 72 h of monitoring are reported in Figure 4a,b. Specifically, data in Figure 4a are relevant to test where a small surface to volume ratio is considered (0.44 cm^2^/cm^3^) whereas, Figure 4b highlights the results for the test where a threefold higher surface to volume ratio (1.3 cm^2^/cm^3^) was taken into account (vide infra).

As can be seen in Figure 4a, the curves show that the two control systems recorded a similar trend and after 24 h the microbial counts achieved high cell loads, accounting in both cases for more than 8 Log CFU/mL. The active film, instead, recorded a lower microbial count after 1 day, compared to both control samples. In Figure 4b, it is possible to infer that the effects of the active film, on the mix of two selected target microorganisms, are more marked. The increase in the surface to volume ratio of active film allowed maintaining low *Pseudomonas* loads for more than 24 h (around 4 Log CFU/mL) compared to the Ctrl* and PHBV* films which reached microbial concentration higher than 8 Log CFU/mL after 1 day.

The effectiveness of the active systems is not surprising, because it is well known from the literature that silver ions are active on many spoilage bacteria or pathogens. Silver is known for its inhibitory effects, due to its ability to reduce the integrity of cellular membranes, deactivation of enzymes and denaturation of DNA molecules [26,27]. In agreement with our study, Dairi et al. [28] investigated the active properties of AgNPs, and observed important antimicrobial effects against pathogenic bacteria and fungi. Kanmani and Rhim [29] observed that AgNPs embedded in a gelatin film exhibited pronounced antimicrobial activity against *L. monocytogenes*. The antimicrobial activity of alginate hydrogels filled with AgNPs was evaluated by Rescignano et al. [30], who showed that active hydrogels inhibited growth of both *E. coli* and *P. aeruginosa* due to direct contact of film with bacteria.

In the current study it is worth noting how the effects of these active systems can be modulated according to the surface/volume ratio taken into account. To better highlight the differences between the two selected surface to volume ratios, the fitting of experimental data was carried out in order to estimate fitting parameters (Table 2) as values with biological meaning [31]. Specifically, A (maximum cell increase at the stationary phase), *μ*_max_ (maximum growth rate), τ (Lag time) and t* (time to achieve 6.0 Log CFU/mL) were calculated. As regards to films with a lower surface/volume ratio, no marked differences were found in terms of A and Lag time in the three samples, whereas the *μ*_max_ value is significantly (*p* < 0.05) lower in the active film than in both control systems. This finding is also highlighted in Figure 4a, because the exponential growth phase begins almost simultaneously in all the samples. Since the maximal growth rate of *Pseudomonas* in AgNPs-PHBV is lower than in the two control systems, the microbial slowdown is evident. The t* value of the active film is reached after 16.8 h of monitoring, whereas it is after 11.5 h (Ctrl) and 12.2 h (PHBV) in the control samples.

When a higher surface/volume ratio was taken into account, more interesting fitting parameters were recorded because the active composite systems were found to be more effective. In particular, the efficacy of the AgNPs was more evident in terms of *μ*_max_, τ and consequently in the t* value (Table 2). Specifically, it is worth noting that in the presence of the active material, microorganisms increased after 17 h of Lag phase, compared to control systems where microbial proliferation appeared just after 7–8 h. Therefore, in the samples with the active film, the microbial load of *Pseudomonas* spp. remained lower than that of the two controls for over 30 h. Statistically significant differences (*p* < 0.05) between samples with and without Ag were also recorded in terms of maximal growth rate. The striking future of fitting data is also the evidence that the t* value of active film was threefold higher than that of both controls.

## 3. Materials and Methods

### 3.1. Materials

Milli-Q water (25 °C, 18.2 MΩ) was used throughout the experiments. Silver sheets (99.99% purity, diameter: 10 mm; nominal thickness: 1 mm) for laser ablation were purchased from GoodFellow Ltd. (Cambridge, UK). Pads for the mechanical polishing of metal targets were obtained from Buehler (Lake Bluff, IL, USA). Isopropanol (IPA, >99.9%, HPLC grade) HNO_3_ (67%, Trace-SELECT^®^ Ultra, for ultratrace analysis), NaCl (Trace-SELECT^®^ Ultra, ≥99.999%, for ultratrace analysis), NaH_2_PO_4_ (Trace-SELECT^®^, ≥99.99%, for trace analysis), and Na_2_HPO_4_ (Trace-SELECT^®^, ≥99.99%, for trace analysis) were purchased from Sigma Aldrich (Milan, Italy). Silver standard for AAS (Ag pure single-element standard, 1000 μg/mL in 2% HNO_3_) was purchased from Perkin Elmer (Milan, Italy). TEM samples were prepared on carbon-coated, 300-mesh, Cu grids purchased from Agar Scientific (Stansted, UK). PHBV thermoplastic resin (>98%, Y1000P) was obtained by EN-MAT^™^ (Manchester, UK) in the form of pellets for thermoforming processes. The molecular weight was about 240 kDa and the content of valeric acid was approximately 3% according to the data sheet [32].

### 3.2. Production of AgNPs by Laser Ablation and Composite Preparation

The experimental procedure used for AgNP production by LASiS was already described elsewhere [15]. In brief, we exploited an ns Q-Switched Nd:YAG Laser (Quantel- BRIO), operating at the fundamental wavelength of 1064 nm, with a pulse frequency of 20 Hz, a pulse energy of 46.5 mJ (fluence 2.33  ±  0.05 mJ/cm^2^), and a nominal pulse duration of 4 ns. The Ag target was fixed to the wall of a 35 × 25 × 30 mm^3^ quartz rectangular cell, filled with 10 mL of IPA. The ablation duration was 60 min.

PHBV films were prepared by melt processing [8] after pre-drying all materials at 60 °C for 12 h before molding. All substrates were prepared using a Colin hot plate press at a temperature of 180 °C with a maximum molding pressure of 5 bar for a time of 5 min. Thermoformed PHBV sheets thickness was measured with manual calipers and resulted in being equal to 4.0 ± 0.2 mm.

Films were cut in 2 × 2 cm^2^ samples for further characterizations and experiments. For composite preparation, each sample was rinsed with IPA and dried under N_2_ flow. After that, 500 µL of freshly prepared colloidal solution were drop-cast, in 5 subsequent aliquots of 100 µL each, and samples were dried in the air and in the dark overnight. Considering a measured colloidal concentration equal to 0.11 ± 0.05 mg/mL, it is possible to assume that each PHBV slide brings about 55 µg of Ag on 2 × 2 cm^2^.

### 3.3. Morphological and Spectroscopic Characterization

The optical absorption spectrum of the ablated colloid was acquired using UV-Vis spectroscopy (Shimadzu UV-1601) in the 250–800 nm range, in quartz Suprasil^®^ cuvettes from Hellma Analytics (Müllheim, Germany).

AgNP morphology was studied by TEM, using a FEI Tecnai 12 instrument (120 kV; filament: LaB_6_). The microscope was calibrated by using the S106 Cross Grating (2160 lines/mm, 3.05 mm) provided by Agar Scientific. A size distribution histogram was obtained after TEM images processing performed manually, through the ImageJ (version 1.8.0) software (Laboratory for Optical Computational Instrumentation, Madison, WI, USA; National Institutes of Health, Bethesda and Rockville, MD, USA).

Chemical characterization of the PHBV composites was obtained by ATR-FTIR analysis on a Spectrum Two FT-IR Spectrometer (PerkinElmer, Milan, Italy) with a resolution of 2 cm^−1^ and with scanning from 4000 to 400 cm^−1^. A number of 32 scans were averaged; background spectra were acquired against air, and spectra baseline subtraction was performed using the instrument software.

### 3.4. Determination of Silver Release

Silver ion release was determined by electrothermal atomic absorption spectroscopy (ETAAS). Modified Ag-PHBV substrates and pristine ones were put in Petri dishes filled with 2 mL of PBS (pH = 6.8, ionic force I = 0.1) solution. This pH resembles the one of real apple juice samples used for antimicrobial experiments. At each sampling time (5 min, 10 min, 30 min, 1 h, 2 h, 4 h, 7 h, 24 h, 30 h), contact solution was sampled and properly diluted with 0.2% HNO_3_ to undergo ETAAS analysis. We used a Perkin Elmer PinAAcle AS900Z instrument, equipped with a silver mono-elemental hollow cathode lamp and a transversely heated graphite furnace. Sample thermal treatment in the graphite furnace was programmed as follows:

- Step 1a: 110 °C in 1 s, hold time 45 s;
- Step 1b: 130 °C in 15 s, hold time 90 s;
- Step 2: 1200 °C in 10 s, hold time 20 s;
- Step 3: 2000 °C in ~0 s, hold time 5 s;
- Step 4: 2450 °C in 1 s, hold time 5 s.

Calibration curve was performed by appropriate dilutions of an Ag standard for AAS It is reported in Figure S2. Release kinetic data were curve fitted by Origin Pro^©2021^ software (Northampton, MA, USA), using a pseudo-first order kinetic model Equation (1).

### 3.5. Antimicrobial Activity of AgNP-PHBV Composites

To test the antimicrobial activity of developed AgNP-PHBV composites, cell growth experiments were carried out by in vitro test. To this aim, two species of *Pseudomonas* spp. isolated from spoiled food, identified as *P. fluorescens* and *P. putida*, were selected as test microorganisms. The two strains were maintained in Plate Count broth (PC, Oxoid, Milan, Italy) at −20 °C with the addition of 30% of glycerol as stock cultures. Prior to the antimicrobial tests, exponentially growing cultures were obtained by allowing each strain to grow in appropriate broth at 25 °C for 24 h. Then, a cocktail of the two strains was prepared by mixing 1% of each culture. The inoculum was prepared by diluting the exponentially growing cultures with sterile saline solution (9 g/L NaCl) to obtain approximately 10^3^ CFU/mL. A direct plate count technique was used to ensure a good degree of reproducibility in the inoculum preparation.

For the in vitro tests, clear fruit juice was chosen as a food simulating system, inoculated with the microbial cocktail, and spread in different tubes with and without films. Two different surface/volume ratios between film and juice were used for comparative purposes. Specifically, a test was carried out using 4 cm^2^ of active composite in 9 mL inoculated juice, and another test was carried out using 8 cm^2^ in 6 mL. As controls, inoculated tubes with bare PHBV and tubes without any polymer were also prepared. All tubes were incubated at 25 °C for 72 h. After 4, 9, 24, 30, 48, 57, and 72 h, aliquots of 1 mL were taken from each tube for microbiological analyses. To avoid modifications in the microbial concentration due to sampling, each tube was used only for a single measure. Each sample was diluted serially with sterile saline solution (9 g/L NaCl) and then plated on a Pseudomonas Agar Base (PAB), modified by adding Pseudomonas CFC selective supplement (autoclaved at 121 °C for 15 min) and finally incubated at 25 °C for 48 h. All analyses were performed twice on two different samples. pH was also measured in each broth, using a pH-meter (Crison, Barcelona, Spain). Two different samples were used for each measurement. In order to quantitatively compare the microbial effectiveness of active and control packaging systems, the following Gompertz Equation, as re-parameterized by Zwietering et al. [25], was fitted to the experimental data:

(2) $$\log N\left(t\right)=\log N_{0\;}-A\cdot exp\left\{-exp\left\{\left[\left(\;\mu_{max}\cdot 2.71\right)\cdot \frac{\tau }{A}\right]+1\right\}\right\}+A\cdot exp\left\{-exp\left\{\left[\left(\mu_{max}\cdot 2.71\right)\cdot \frac{\tau -t}{A}\right]+1\right\}\right\}$$

where *N*(*t*) is the viable cell concentration at time *t*, *N*_0_ is the initial value of cell concentration, *A* is related to the difference between decimal logarithm of the initial value of cell concentration and the decimal logarithm of the bacterial growth attained at the stationary phase, *μ*_max_ is the maximum specific growth rate, *τ* is the Lag time, and t* is the time to reach the concentration of 6 Log CFU/mL intended as the microbial count when spoilage defects start to appear on food [13]. To determine whether significant differences (*p* < 0.05) existed among the fitting parameters, the one-way variance analysis (ANOVA) and Duncan’s multiple range test with the option of homogeneous groups were used by means of STATISTICA 7.1 for Windows (StatSoft Inc., Tulsa, OK, USA).

## 4. Conclusions

Active PHBV was obtained by modifying its surface with laser-ablated AgNPs. The nanomaterial was characterized by UV-Vis and TEM, in order to study its optical and morphological properties; afterwards, we developed a simple protocol to modify PHBV without altering its physicochemical properties. In fact, ATR-IR spectra did not show any modification in the main IR bands relevant to the polymeric matrix. The silver release analysis was used to theorize the antimicrobial action of the novel nanomaterial. AgNP-PHBV films were also tested on inoculated apple juice samples, where they were shown to be active against microbial proliferation. An increased antimicrobial effectiveness, in particular in terms of maximum growth rate, Lag time, and time to achieve the maximum cell load, was recorded by increasing the surface to volume ratio. Due to the recorded results, the proposed laser ablation synthesis is a promising technique to develop biodegradable active polymers intended for food packaging applications.

## Figures and Tables

**Table 1:** Spectral IR regions of interest. ν indicates symmetrical (s), asymmetrical (a) stretching; δ represents vibrational distortions in conjugated molecules. Uncertainty on positions is 2 cm^−1^.

Attributions	Spectral Regions (cm^−1^)
ν_s_ (CH, CH_2_), ν_a_ (CH, CH_2_)	2975–2930
ν_s_ (C=O)	1718
ν_s_ (C-C)	1450–1380
ν_s_ (C-O)	1130
δ (C-C)	970–824

**Table 2:** Values of parameters calculated by fitting experimental data with Equation (2).

Samples	A (log CFU/mL)	*μ*_max_ (Δlog CFU/mL/h)	λ (h)	t* (h)
Ctrl	5.9 ± 0.06 ^a^	0.60 ± 0.08^c^	6.8 ± 0.35 ^b^	11.55 ± 0.36 ^a^
Bare PHBV	6.1 ± 0.09 ^a^	0.43 ± 0.02 ^b^	5.2 ± 0.39 ^a^	12.2 ± 0.27 ^a^
AgNPs-PHBV	6.1 ± 0.13 ^a^	0.29 ± 0.01 ^a^	6.4 ± 0.82 ^b^	16.8 ± 0.43 ^b^
Ctrl*	5.4 ± 0.12 ^A^	0.51 ± 0.1 ^B^	7.2 ± 0.56 ^A^	13.28 ± 0.9 ^A^
Bare PHBV*	5.4 ± 0.28 ^A^	0.79 ± 0.25 ^B^	8.0 ± 0.63 ^A^	12.12 ± 0.52 ^A^
AgNPs-PHBV*	5.9 ± 0.65 ^A^	0.18 ± 0.04 ^A^	17.23 ± 3.86 ^B^	37.33 ± 2.52 ^B^

Values marked with different superscript letters (a,c) in the same column are significantly different (*p* < 0.05). Ctrl: bare inoculated sample; PHBV: inoculated sample with PHBV (4 cm^2^); AgNPs-PHBV: inoculated sample with AgNPs-PHBV. (b) Ctrl*: bare inoculated sample; PHBV*: inoculated sample with PHB (8 cm^2^); AgNPs-PHBV*: inoculated sample with AgNPs-PHBV (8 cm^2^).

## Data Availability

The data is available in this manuscript.

## Supplementary Materials

Supplementary materials can be found at https://www.mdpi.com/article/10.3390/antibiotics10070760/s1. **Figure S1**: UV-Vis spectrum of AgNPs synthesized by LASiS in IPA. Figure S2: Silver calibration curve for AAS analysis. R^2^ = 0.99936. Figure S3: TEM micrographs assessing the possible release of entire Ag nanoparticles within 30 h of contact with PBS.
