# Supplementary material for: A New Nanocomposite Packaging Based on LASiS-Generated AgNPs for the Preservation of Apple Juice
Source: Antibiotics (Basel). 2021 Jun 22;10(7):760. doi: 10.3390/antibiotics10070760 (PMC8300681; doi:10.3390/antibiotics10070760)
Supplement: Supplementary file 1 [file antibiotics-10-00760-s001.zip › antibiotics-1257244-supplementary.pdf]

Supplementary material

A new nanocomposite packaging based on LASiS-generated AgNPs for the preservation of apple juice

Maria Chiara Sportelli [1,2], Antonio Ancona [2,3], Annalisa Volpe [2,3], Caterina Gaudiuso [2,3], Valentina Lavicita [4], Valerio Miceli [5], Amalia Conte [4], Matteo Alessandro Del Nobile [4], and Nicola Cioffi [1,*]

[1] Chemistry Department, University of Bari, Via Orabona, 4-70126 Bari, Italy; maria.sportelli@uniba.it, nicola.cioffi@uniba.it.
[2] IFN-CNR, Physics Department, University of Bari, Via Amendola, 173-70126 Bari, Italy;
[3] Physics Department, University of Bari, Via Orabona, 4-70126 Bari, Italy; antonio.ancona@uniba.it, annalisa.volpe@uniba.it, caterina.gaudiuso@uniba.it.
[4] Department of Agricultural Sciences, Food and Environment, University of Foggia, Via Napoli 25-71122 Foggia, Italy; valentina.lacivita@unifg.it, amalia.conte@unifg.it, matteo.delnobile@unifg.it.
[5] ENEA Research Center, BIOAG division - ss Appia km 700 – 72100 Brindisi, Italy; valerio.miceli@enea.it.
Correspondence: nicola.cioffi@uniba.it; Tel.: +39 080 5442020.

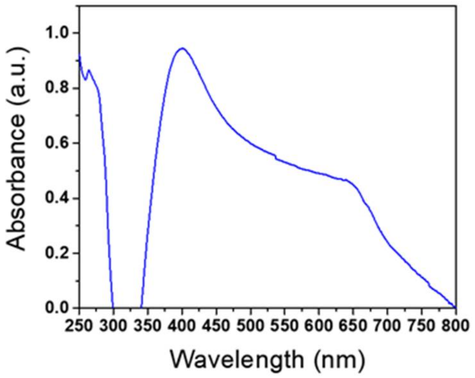

Figure S1. UV-Vis spectrum of AgNPs synthesized by LASiS in IPA.

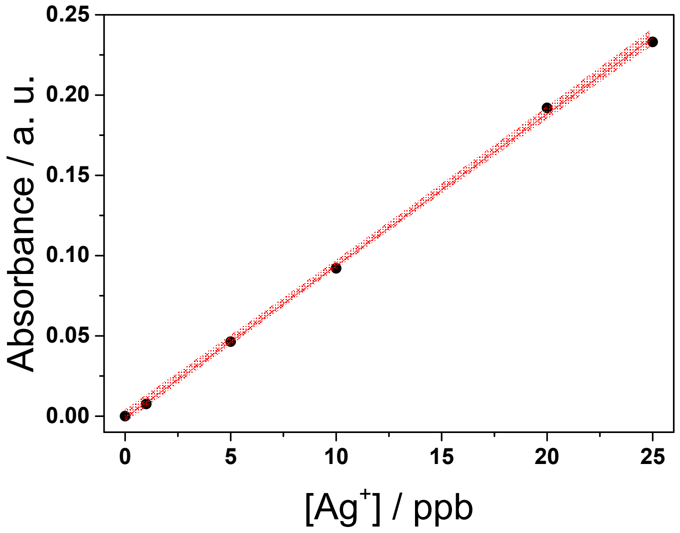

Figure S2. Silver calibration curve for AAS analysis. R^2 = 0.99936.

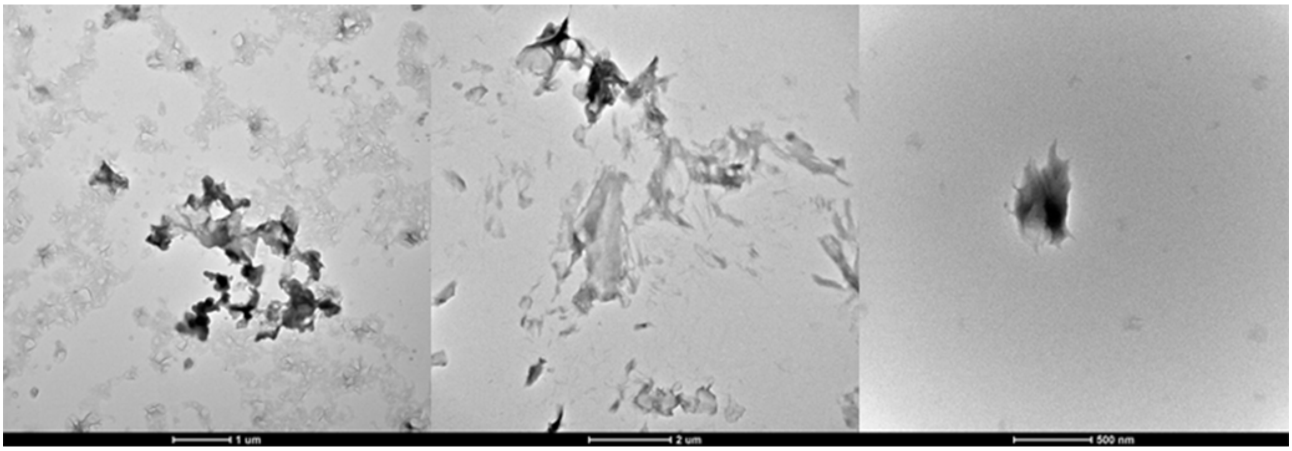

Figure S3: TEM micrographs assessing the possible release of entire Ag nanoparticles within 30 h of contact with PBS.
